# Evaluation of liver enzyme elevations and hepatotoxicity in patients treated with checkpoint inhibitor immunotherapy

**DOI:** 10.1371/journal.pone.0253070

**Published:** 2021-06-11

**Authors:** Morven Cunningham, Marco Iafolla, Yada Kanjanapan, Orlando Cerocchi, Marcus Butler, Lillian L. Siu, Philippe L. Bedard, Kendra Ross, Bettina Hansen, Anna Spreafico, Jordan J. Feld

**Affiliations:** 1 Toronto Centre for Liver Disease, University Health Network, Toronto, Canada; 2 Princess Margaret Cancer Centre, University Health Network, Toronto, Canada; University of Navarra School of Medicine and Center for Applied Medical Research (CIMA), SPAIN

## Abstract

**Background and aims:**

Immune checkpoint inhibitors (ICI) are increasingly used in cancer therapy. Elevated liver enzymes frequently occur in patients treated with ICI but evaluation is poorly described. We sought to better understand causes of liver enzyme elevation, investigation and management.

**Methods:**

Patients treated with anti-PD-1, PDL-1 or CTLA-4 therapy in Phase I/II clinical trials between August 2012 and December 2018 were included. Clinical records of patients with significant liver enzyme elevations were retrospectively reviewed.

**Results:**

Of 470 ICI-treated patients, liver enzyme elevation occurred in 102 (21.6%), attributed to disease progression (56; 54.9%), other drugs/toxins (7; 6.9%), other causes (22; 21.6%) and ICI immunotoxicity (17; 16.7%; 3.6% of total cohort). Immunotoxicity was associated with higher peak ALT than other causes of enzyme elevation (*N* = 17; *M* = 217, 95% CI 145–324 for immunotoxicity, *N* = 103; *M* = 74, 95% CI 59–92 for other causes; ratio of means 0.34, 95% CI 0.19–0.60, p = <0.001) and higher ALT:AST ratio (M = 1.27, 95% CI 0.78–2.06 for immunotoxicity, M = 0.69, 95% CI 0.59–0.80 for other causes, ratio of means 0.54, 95% CI 0.36–0.82, p = 0.004). Immunotoxicity was more often seen in patients with prior CPI exposure (41.2% of immunotoxicity vs 15.9% of patients without, p = 0.01), anti-CTLA-4 –containing ICI treatments (29.4% of immunotoxicity vs 6.8% of patients without, p = <0.001) and other organ immunotoxicity (76.5% of immunotoxicity vs 19.2% of patients without, p = <0.001). Cause for enzyme elevation was established in most patients after non-invasive investigation. Liver biopsy was reserved for four patients with atypical treatment response.

**Conclusions:**

Liver enzyme elevation is common in patients receiving ICI, but often has a cause other than immunotoxicity. A biochemical signature with higher ALT and ALT/AST ratio, a history of prior ICI exposure and other organ immunotoxicities may help to identify patients at a higher likelihood of immunotoxicity. Liver biopsy can be safely deferred in most patients. We propose an approach to diagnostic evaluation in patients with liver enzyme elevations following ICI exposure.

## Introduction

Immune checkpoint inhibitors (ICI) are a novel class of anticancer therapy which enhance immunological response against tumour cells, and are approved for treatment of several malignancies. Their use is associated with a distinct profile of side effects, termed immune-related adverse events (irAE), which cause immune-mediated organ damage [1]. Liver immunotoxicity, manifesting as liver enzyme elevation, is well recognized with use of ICI with varying incidence reported by class. Anti-CTLA-4 drugs appear to be associated with a higher risk for hepatotoxicity than PD-1 or PD-L1 inhibitors [2, 3]. Combination therapy may have a synergistic effect on the risk of hepatotoxicity—any grade ALT elevation was reported in 3.8% of nivolumab-treated patients, 3.9% of patients who received ipilimumab, but 17.6% of patients treated with both agents [4].

The best approach to evaluation and management of liver enzyme elevations in ICI-treated patients remains uncertain. Current literature is sparse on how frequently liver injury during ICI therapy is due to immunotoxicity versus other aetiologies, although a recent report suggests that progression of underlying malignancy is a far more common cause for enzyme elevation than immunotoxicity in patients treated with Pembrolizumab [5]. Assessment may be complicated by widespread use of Common Terminology for Clinical Adverse Events Criteria (CTCAE), widely used to identify and stratify liver enzyme elevations in patients treated with ICI [6]. These differ from criteria more familiar to hepatologists in management of drug-induced liver injury (DILI) [7, 8]. No management protocol has yet been formally evaluated in management of liver immunotoxicity, so optimal strategies for diagnosis, assessment and treatment remain unclear.

The aim of this study was to describe clinical characteristics of patients who experience clinically significant liver enzyme elevations during ICI. Specifically, this study aimed to characterize frequency and severity of liver enzyme elevations in ICI-exposed patients, diagnostic evaluation undertaken, proportion where an alternate cause for enzyme elevation was identified and explore clinical features of patients with ICI-related hepatotoxicity.

## Methods

This was a retrospective, single centre study conducted at a tertiary cancer centre (Princess Margaret Cancer Centre, Toronto, Canada). All patients treated with immunotherapy in a Phase I or II trial since August 2012 were recorded in the Princess Margaret Tumour Immunotherapy Programme (TIP) Database, with extensive clinical characterisation.

Inclusion criteria for this study were initiation of immunotherapy between August 2012 and December 2018; treatment with anti-PD-1, anti-PD-L1 and/or anti-CTLA-4 agents as monotherapy or combined with other immunotherapy; and laboratory values (bilirubin, ALT, AST and ALP) available during and after treatment on an electronic patient record. To reduce heterogeneity, patients treated exclusively with non-ICI immunotherapies were excluded.

The TIP database was used to identify patients who met inclusion/exclusion criteria. Electronic patient records were searched for ≥Grade (G)2 elevation in bilirubin, ALT, AST or ALP during the study period. As CTCAE (version 4.03) had been used contemporaneously, these criteria were used to stratify enzyme elevations (S1 Table). Enzyme elevation ≥G2 was deemed clinically significant and used for study inclusion as most trial protocols and clinical guidelines recommend holding ICI therapy and consideration of immunosuppression at this threshold [9, 10]. For patients with baseline enzyme elevation, updated CTCAE criteria (version 5.0) were retrospectively applied to grade further liver enzyme elevations after ICI treatment (S1 Table). Dates of bilirubin, ALT, AST or ALP elevation were cross-referenced with dates of ICI therapy. Patients in whom liver enzyme elevation pre-dated ICI therapy were excluded from further analysis.

Clinical records were reviewed in detail for the evaluation performed and final diagnosis, as recorded by the treating physician. In patients diagnosed with liver immunotoxicity, additional information was collected on treatment, time to resolution and outcome of any further ICI exposure. The Roussel Uclaf Causality Assessment Method (RUCAM) is a validated tool used to estimate likelihood of drug causality in liver injury [11]. This was applied retrospectively to patients with enzyme elevations during or after ICI exposure. The following interpretations of RUCAM scores were used: <0, drug is “excluded”; 1–2, “unlikely”; 3–5, “possible”; 6–8, “probable”; and >8, “highly probable” cause for liver injury. R ratio was calculated to classify the pattern of liver injury (R = (ALT/ALT ULN)/(ALP/ALP ULN)). R >5.0 was classed as hepatocellular, R <2.0 as cholestatic, and R = 2.0–5.0 as mixed pattern [11].

The study protocol conforms to the 1975 Declaration of Helsinki. TIP database and study protocol were reviewed and approved by University Health Network Research Ethics Board (UHN REB; TIP database reference 15–9269; study protocol reference 18–5547). Patient identifiable information was accessible to the investigators performing the initial chart review (MC, MI, YK). Subsequently, participants were identified by study number only and all data were anonymised for further analysis. Electronic medical records were accessed for this study between January 2018 and January 2020. Written, informed consent was obtained from each patient included in the TIP database. As this was a retrospective study, and many patients had since died, further patient consent was not obtained. This approach was approved by UHN REB.

### Statistical analyses

Data were grouped according to presence or absence of enzyme elevation, then according to diagnosis (immunotoxicity or other). Normality was assessed using D’Agostino-Pearson test for Normal distributions. None of the study data fitted a Normal distribution. Non-Normally distributed, continuous variables were log-transformed prior to further analysis. Student’s t-test was then used to assess for statistical significance between groups. Back-transformed results were expressed as geometric mean (M) and 95% confidence interval (95% CI). The ratio of the geometric means is the back-transformed difference of the means of the logs of the two samples. For non-parametric, semi-quantitative data, Mann-Whitney U test was used to assess for statistically significant differences between groups. For non-continuous variables, Chi squared or Fisher’s exact tests were used. Differences in cancer progression and overall survival were assessed using Cox proportional hazards regression analysis, with liver immunotoxicity included as a time-dependent co-variate. Other baseline variables included age, sex, primary cancer and type of immunotherapy. Progression was defined by radiological or clinical assessment, as per trial protocols. All tests were two-sided and a *p* value of <0.05 considered statistically significant. Statistical analyses were performed using MedCalc statistical software (version 17.9.7, Ostend, Belgium) and IBM SPSS (version 25.0; NY, USA).

## Results

### Patient cohort

**Fig 1:**
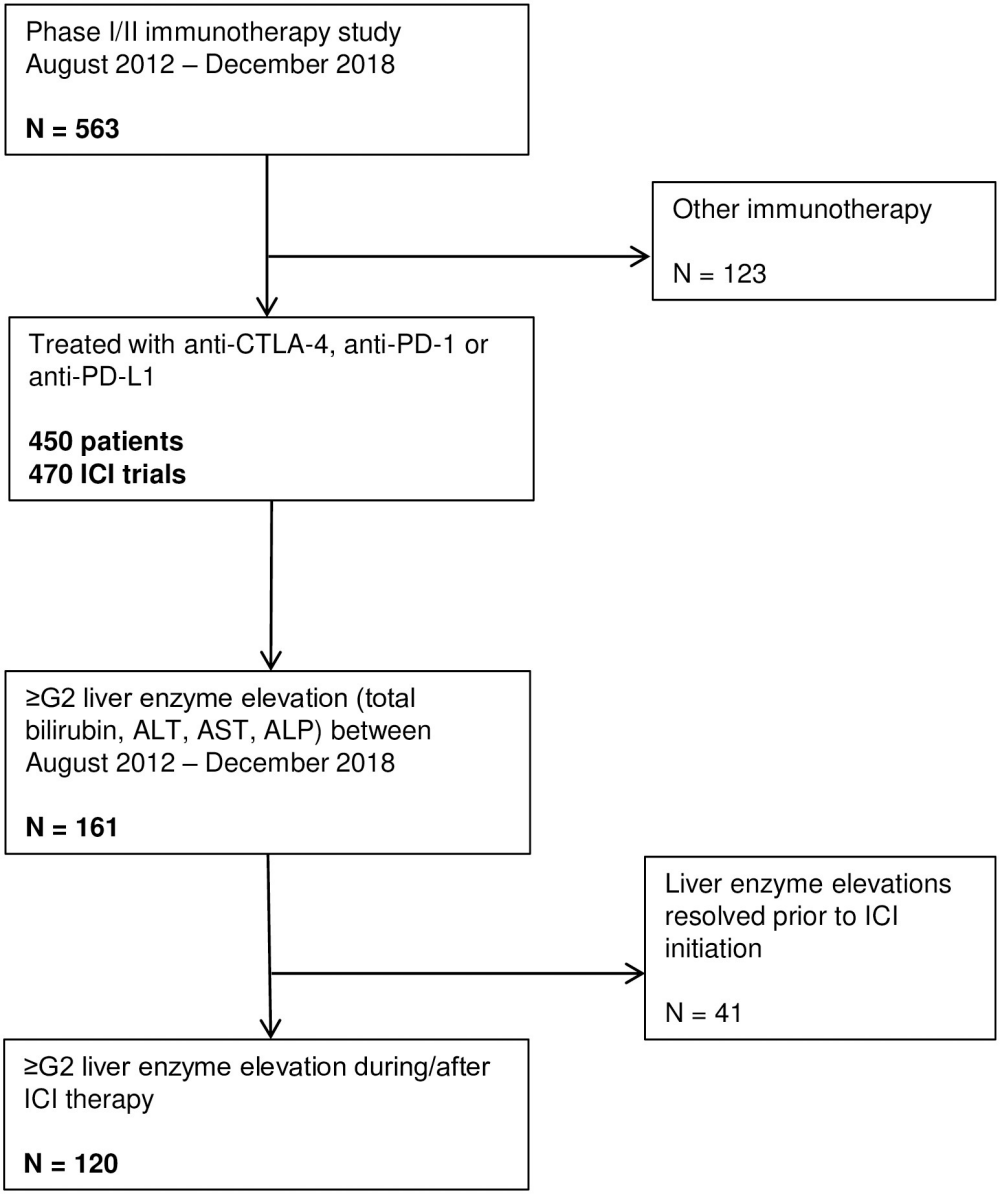
Flow diagram illustrating identification of patients with liver enzyme elevation for inclusion in the study. ICI, checkpoint inhibitor; ALT, alanine aminotransferase; ALP, alkaline phosphatase; PD-1, programmed death protein 1; CTLA-4, cytotoxic T-lymphocyte-associated protein 4; PD-L1, programmed death-ligand 1; ALP, alkaline phosphatase; ALT, alanine aminotransferase; AST, aspartate aminotransferase.

Between August 2012 and December 2018, 573 patients were treated with immunotherapy within a Phase 1 or 2 clinical trial. We excluded 123 patients who did not receive anti-PD-1, anti-PD-L1 or anti-CTLA-4 therapy, leaving 450 patients treated with these agents, as monotherapy or in combination with other immunotherapy, in 470 trials (some patients were treated in more than one trial; Fig 1). Most (313; 66.6%) were treated with anti-PD-1 therapy; 116 (24.7%) with anti-PD-L1 and a minority received anti-CTLA-4 (6; 1.3%) or combination CPI (35; 7.5%; S2 Table). The most common primary malignancies were melanoma (72; 16.0%), head and neck (71; 15.8%), genitourinary (70; 15.6%); lung (60; 13.3%) or gastrointestinal (59; 13.1%), although many primary malignancies were represented (S3 Table).

### Liver enzyme elevations

Reviewing electronic records, 161 patients (34.3%) had a clinically significant (≥G2) bilirubin, ALT, AST or ALP elevation during the study period. After cross-referencing with dates of therapy, 41 were excluded as liver enzyme elevation had resolved prior to CPI, leaving 120 patients (25.5%) with ≥G2 enzyme elevation during or after CPI therapy (Fig 1). Enzyme elevation occurred during ICI therapy in 69 patients (57.5%; median 42 days (range 2–483) from first dose) and after ICI therapy in 51 (42.5%; median 109 days (range 2–760) from last dose). Pattern of enzyme elevation was hepatocellular (R > 5.0) in 17 (14.2%), cholestatic (R < 2.0) in 84 (70.0%), and mixed (R = 2.0–5.0) in 19 (15.8%). Severe (G3/4) AST/ALT elevation occurred in 44 patients (9.4% of all ICI treated patients), ALP elevation in 26 (5.5%) and bilirubin elevation in 22 (4.7%). There was no significant difference in likelihood of liver enzyme elevation amongst patients treated with anti-PD-1, anti-PD-L1, anti-PD-1/PD-L1 combination, or anti-CTLA-based therapy (*X*^*2*^ (3, N = 470) = 5.66, p = 0.13).

#### Diagnostic evaluation

In 17 patients, liver enzyme elevation either had a clear trigger (e.g. surgery), resolved rapidly or was associated with clinical features of end-stage cancer, and no further investigations were pursued. Of the remaining 103 patients, all had liver imaging within a four-week period either before or after enzyme elevation. HBV/HCV serology results were available for 96 patients (93.2%); 15 (14.6%) tested after enzyme elevation and 81 (78.6%) prior to commencement of ICI, although this may underestimate the extent of testing as data were limited to results from our centre. Autoimmune serologies were checked in 14 (13.6%). Four patients were referred to a hepatologist and had a liver biopsy (3.3%). Reasons for referral were presumed immunotoxicity with failure to respond to either holding ICI or corticosteroids (N = 3), and rebound ALT/AST after corticosteroid taper (N = 1). Additional investigations in patients seen by a hepatologist were extended virology screening (including HAV, HEV, EBV and CMV), caeruloplasmin, and liver biopsy. A diagnosis of immunotoxicity was confirmed in two patients. Alternate diagnoses of mushroom toxicity and non-alcoholic fatty liver disease were made in the remaining two patients.

**Fig 2:**
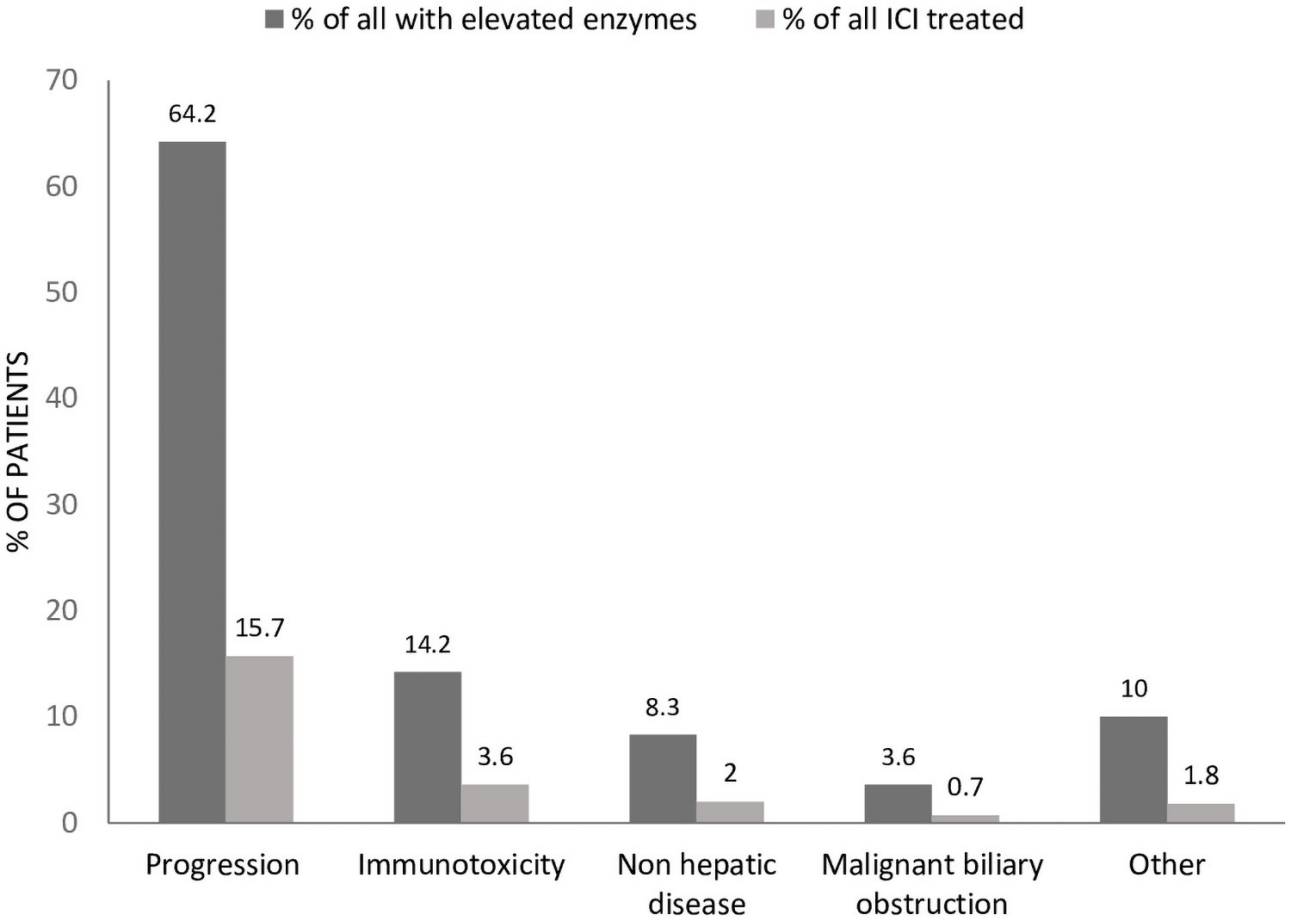
Final diagnoses of liver enzyme elevation after exposure to checkpoint inhibitor immunotherapy (ICI). Range and frequency of diagnoses that were made by the treating clinician, based on clinical assessment and results of investigations performed. Dark grey bars show patients with each diagnosis as a percentage of all patients with elevated lever enzymes after ICI exposure. Light grey bars show patients with each diagnosis as a percentage of all patients treated with ICI.

The final diagnosis for enzyme elevation, as documented in the clinical record by the treating clinician, was ICI-associated immunotoxicity in 17 patients (14.2% of those with enzyme elevations; 3.6% of all ICI exposures), disease progression in 77 (64.2%), non-hepatic disease in 10 (8.3%), malignant biliary obstruction in 4 (3.6%) and other causes (including other drug/toxins, non-malignant biliary obstruction and fatty liver disease) in 12 (10%; Fig 2).

### Liver immunotoxicity

Following investigation, 17 patients had a final diagnosis of ICI-associated immunotoxicity. Immunotoxicity occurred during ICI therapy in 13 patients (median 44 days (range 23–295) from first dose) and after ICI therapy in four (median 30 days (range 21–49) from last dose). To corroborate the contemporaneous diagnosis of immunotoxicity, RUCAM scores were calculated retrospectively for all patients with elevated liver enzymes following ICI exposure. RUCAM has not been specifically validated in the indirect type of liver injury associated with ICI [11, 12], although RUCAM and DILIN causality assessments correlate well for patients with ICI-associated immunotoxicity [5]. Although limited in many cases by lack of full investigations to exclude other causes of liver disease, RUCAM scores were significantly higher in patients who had been diagnosed with immunotoxicity than those given an alternate diagnosis, supporting a causative role for ICI (median RUCAM score for patients diagnosed with immunotoxicity was 7 (N = 17; range 4–9), vs 1 (N = 103; range -4–3) for patients diagnosed with other causes of enzyme elevation, Mann-Whitney *U* = 39, *z* = 6.52, p <0.0001; S1 Fig). All patients diagnosed with immunotoxicity responded to ICI withdrawal +/- corticosteroids, further supporting the likelihood of immunotoxicity. A detailed description of clinical characteristics of patients diagnosed with immunotoxicity is given in S4 Table.

#### Biochemical characteristics of liver immunotoxicity

Pattern of liver enzyme elevation in patients diagnosed with immunotoxicity was mixed in eight (47.1%), hepatocellular in six (35.3%) and cholestatic in three (17.6%). Differences in liver enzyme elevations between patients diagnosed with liver immunotoxicity and other causes are described in Table 1. Patients diagnosed with immunotoxicity were more likely to have a severe (G3 or 4) ALT/AST elevation and higher ALT:AST ratio than patients with enzyme elevations from other causes. Conversely, patients with liver immunotoxicity had lower peak bilirubin and ALP than patients with enzyme elevations from other causes.

**Table 1:** Laboratory values and checkpoint inhibitor (ICI) therapy exposure in patients with liver enzyme elevations (LEE) attributed to immunotoxicity compared with other causes.

	Liver immunotoxicity (N = 17)	Rest of LEE cohort (N = 103)	P value	Ratio of means (95% CI)
G3/4 ALT/AST elevation (N, %)	13 (76.5)	31 (30.1)	<0.001	
Peak ALT (*M* (95% CI))	217 (145–324)	74 (59–92)	<0.001	0.34 (0.19–0.60)
Peak AST (*M* (95% CI))	170 (116–252)	108 (90–129)	0.053	0.63 (0.40–1.0)
ALT/AST ratio (*M* (95% CI))	1.27 (0.79–2.06)	0.67 (0.59–0.80)	0.004	0.54 (0.36–0.82)
≥G2 Bilirubin elevation (N, %)	2 (11.8)	43 (41.7)	0.015	
Peak bilirubin (*M* (95% CI))	12 (9–17)	25 (20–30)	0.008	2.0 (1.2–3.4)
≥G2 ALP elevation (N, %)	4 (23.5)	64 (62.1)	0.004	
Peak ALP (*M* (95% CI))	174 (115–265)	335 (285–395)	0.003	1.92 (1.25–2.97)
Time on ICI prior to LEE (days; *M* (95% CI))	79 (47–134)	77 (65–91)	0.891	0.97 (0.61–1.53)
ICI (N, %): PD-1	11 (64.7)	62 (60.2)		
PD-L1	1 (5.9)	30 (29.1)		
CTLA-4	3 (17.6)	0		
Combination CPI	1 (5.6)	11 (10.7)		
Blinded	1 (5.6)	0		

ALT, alanine aminotransferase; AST, aspartate aminotransferase; ALP, alkaline phosphatase; PD-1, programmed death protein 1; PD-L1, programmed death ligand 1; CTLA-4, cytotoxic T-lymphocyte-associated protein 4; *M* = geometric mean.

#### Risks for liver immunotoxicity

A comparison between patients with liver immunotoxicity and all other patients treated with ICI is shown in Table 2. Patients with liver immunotoxicity were slightly younger than patients without, but there were no significant differences in sex, presence of liver metastases or exposure to single vs combination ICI between the two groups. Liver immunotoxicity was more frequent in patients with prior ICI exposure (41.2% of patients with immunotoxicity vs 15.9% of patients without, p = 0.01), although the duration of current ICI therapy, and the interval from prior ICI therapy were not associated with increased risk of liver immunotoxicity. Liver immunotoxicity was also more common in patients receiving an anti-CTLA-4 –based treatment regimen, than patients receiving anti-PD-1, anti-PD-L1, or anti-PD-1/PD-L1 combinations (13.9% of patients receiving anti-CTLA-4 vs 2.8% of patients receiving anti-PD-1/PD-L1, p = <0.001). No patient with liver immunotoxicity and prior ICI exposure had experienced significant irAE during previous ICI treatment. Liver immunotoxicity was more common in patients who experienced irAE affecting other organs (76.5% of patients with immunotoxicity vs 20.1% without, p = <0.001). Other organ irAE tended to occur before (N = 8) or concurrent with (N = 7) onset of liver immunotoxicity, rather than after (N = 3; S5 Table).

**Table 2:** Comparison between patients diagnosed with liver immunotoxicity and patients exposed to checkpoint inhibitor (ICI) therapy who did not develop immunotoxicity.

	With liver immunotoxicity (N = 17)	Without liver immunotoxicity (N = 453)	*p* value	Ratio of means (95% CI)
Age (years; *M* (95% CI)	47.9 (39.3–58.4)	57.0 (55.7–58.4)	0.006	1.19 (1.05–1.35)
Sex (M:F)	9:8	231:202	1.000	
Liver metastases (N, %)	4 (23.5)	170 (37.8)	0.216	
Single agent ICI (N, %)	13 (76.5)	418 (92.3)	1.000	
Anti-CTLA-4 –containing regimen (N, %)	5 (29.4)	31 (6.8)	<0.001	
Duration of ICI (days; *M* (95% CI))	106 (59–188)	92 (84–101)	0.573	0.87 (0.54–1.40)
Prior ICI therapy (N, %)	7 (41.2)	72 (15.9)	0.014	
Interval between ICI exposures (days;*M* (95% CI))	96 (21–445)	106 (85–132)	0.792	1.11 (0.51–2.43)
Other irAE (N, %)	13 (76.5)	89 (21.1)	<0.001	

irAE, immune-related adverse event; CTLA-4, cytotoxic T-lymphocyte-associated protein 4; *M*, geometric mean.

### Management of elevated liver enzymes and immunotoxicity

**Fig 3:**
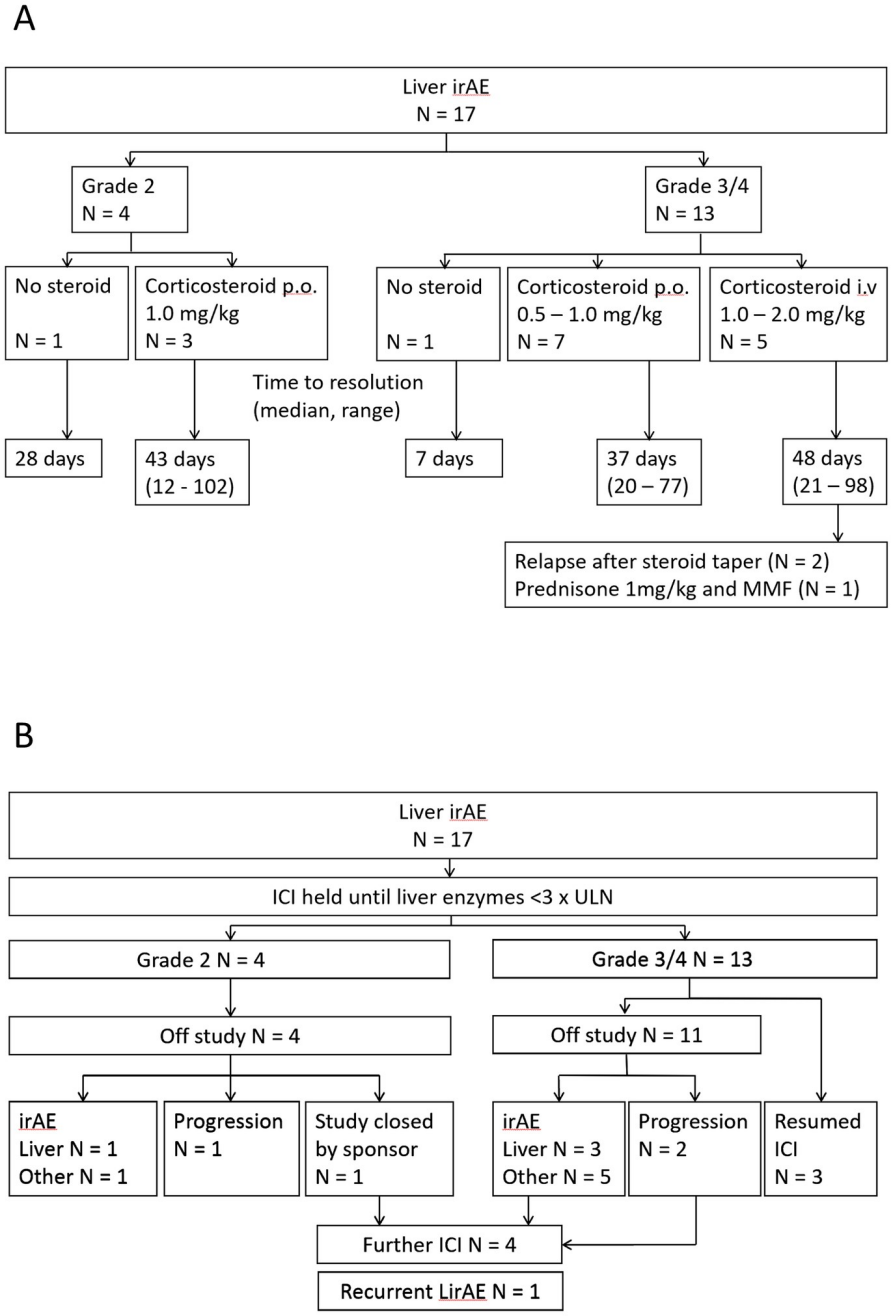
Management of patients diagnosed with liver immunotoxicity due to checkpoint inhibitor (ICI) immunotherapy, according to severity of enzyme elevation. **A**, duration and route of immunosuppressive therapy and time to resolution of elevated liver enzymes. **B**, management of ICI therapy following diagnosis of liver immunotoxicity. ULN, upper limit of normal; irAE, immune-related adverse event; LirAE, liver immune-related adverse event; MMF, mycophenolate mofetil.

Most patients diagnosed with liver immunotoxicity were treated with corticosteroids (Fig 3A; S4 Table). Two patients did not receive corticosteroids, due to either stipulations in the study protocol, or spontaneous improvement. Dose and route of corticosteroid was at the discretion of the treating clinician, guided by specific trial protocols. One patient developed steroid-related side effects requiring introduction of MMF. In all patients, liver enzymes normalized, after median 37 days (range 7–102). ICI therapy was withheld until liver enzymes returned to ≤G1 elevation, on ≤10mg prednisone (Fig 3B). Fourteen patients discontinued ICI, due to liver immunotoxicity (N = 4); other immunotoxicity (N = 6); disease progression (N = 3); and closure of the study (N = 1). Seven patients were subsequently rechallenged with ICI. Only one patient experienced recurrent liver immunotoxicity, G2 severity and successfully managed with oral corticosteroids.

In six patients, enzyme elevations were initially treated as immunotoxicity until investigations identified an alternate diagnosis. In these patients, median duration of ICI hold was 14 days (range 7–28). One patient was presumptively treated with corticosteroids before an alternate diagnosis was made (duration of corticosteroid exposure, 21 days).

### Outcomes

During follow up (median 7.8 months, range 0.2–72.6), 399 patients (88.7%) experienced disease progression and 274 patients (60.9%) died. No patient died from complications related to liver immunotoxicity. In a multivariate analysis including age, sex, type of ICI therapy and cancer type, only cancer type emerged as a significant association with both progression and overall survival, with melanoma associated with the best outcomes. Liver immunotoxicity showed a trend towards reduced cancer progression, although this did not achieve statistical significance (9 patients (52.9%) with immunotoxicity vs. 390 (86.1%) without; HR 0.51; 95% CI 0.23–1.16; p = 0.08). Amongst patients with melanoma, there was a significant association between liver immunotoxicity and reduced progression which persisted in multivariate analysis (2 patients (25.0%) with immunotoxicity vs. 59 (80.8%) without; HR 0.25; CI 0.05–1.17; p = 0.04). This disparity may reflect variation in efficacy of ICI therapy amongst different primary cancers [13], whereby inclusion of patients where ICI was minimally effective dilutes the impact of an association between immunotoxicity and progression. However, the very small number of events limits this analysis and further validation of the hypothesis is needed. There was no significant association between overall survival and liver immunotoxicity in the whole cohort, or the subgroup of patients with melanoma.

## Discussion

In this study, we demonstrated that liver enzyme elevations are common in patients treated with ICI therapy, affecting 25.5% of our cohort. Others have reported similar findings, although descriptions of evaluation or causation have been limited [2, 14]. In keeping with a recent study reporting that cancer progression was the commonest cause for enzyme elevation in Pembrolizumab treated patients [5], in our cohort, treated with a range of ICI, we found that most liver enzyme elevations (85.8%) had a cause other than ICI-associated immunotoxicity. Many were due to progression of intrahepatic metastatic disease, although in almost half of the patients in our series a variety of other causes were diagnosed. Incidence of liver enzyme elevation attributed to immunotoxicity in our study (3.6%) was comparable with other published data [1, 4, 15, 16].

Early and accurate diagnosis of cause of liver enzyme elevations in patients receiving ICI is important to avoid potentially unnecessary interruptions in ICI therapy and exposure to high dose corticosteroids. We found that ICI treatment was interrupted due to enzyme elevations in only 17 patients, mainly due to same-day availability of diagnostic testing which allowed rapid diagnosis of the cause for enzyme elevations. Just four patients with ICI treatment interruption ultimately had a diagnosis other than immunotoxicity, and only one patient commenced corticosteroid treatment, subsequently stopped due to an alternate diagnosis. In the absence of a specific biomarker, diagnosis of liver immunotoxicity remains largely a diagnosis of exclusion. Consistent with other reports, we found that most patients diagnosed with immunotoxicity showed a hepatocellular or mixed pattern of enzyme elevation [5, 17]. We observed that patients with immunotoxicity were more likely to have higher (Grade 3) ALT/AST elevation and higher ALT:AST ratio than patients with other causes for enzyme elevation. Similar to observations by others, we observed that ICI-mediated hepatotoxicity was associated with other organ immunotoxicities [16, 18]. We noted an increased risk of liver immunotoxicity in patients treated with anti-CTLA-4—containing regimens, compared with other classes of ICI. Most patients in this study received anti-CTLA-4 as combination therapy, and combining anti-CTLA-4/PD-1 is recognised to be associated with an increased risk for liver immunotoxicity [4]. We did not, however, observe an increased risk of immunotoxicity with other ICI combination therapies. We found an increased risk of liver immunotoxicity in patients with prior ICI exposure, which may perhaps reflect activating effects on the immune system by prior ICI [4, 19]. We did not find an association between interval between ICI exposures and immunotoxicity in our cohort, but pre-specified washout periods defined in trial protocols may have biased this result.

The role of liver biopsy in diagnosing and managing ICI-associated hepatotoxicity remains uncertain. A recent retrospective series of ICI-treated patients with liver enzyme elevations found that histological features are not associated with response to corticosteroids or need for second line immunosuppression; however, this study was limited by heterogeneity of causes of liver injury and probable selection bias [17]. In most cases in our series, cause of enzyme elevation was diagnosed after non-invasive investigations. Biopsy was reserved for a few patients with suspected immunotoxicity, who failed to respond to ICI withdrawal/corticosteroids. Biopsy identified alternate diagnoses in two patients, and confirmed immunotoxicity in a third, who then successfully transitioned to second line immunosuppression. Arguments against biopsy include delay in starting appropriate therapy, risking progression in liver toxicity and impact on consideration for subsequent immunotherapy. Although the numbers in this study are small, our findings support a position where biopsy can be deferred in the majority of patients, but should be considered if a timely response to treatment is not seen.

**Fig 4:**
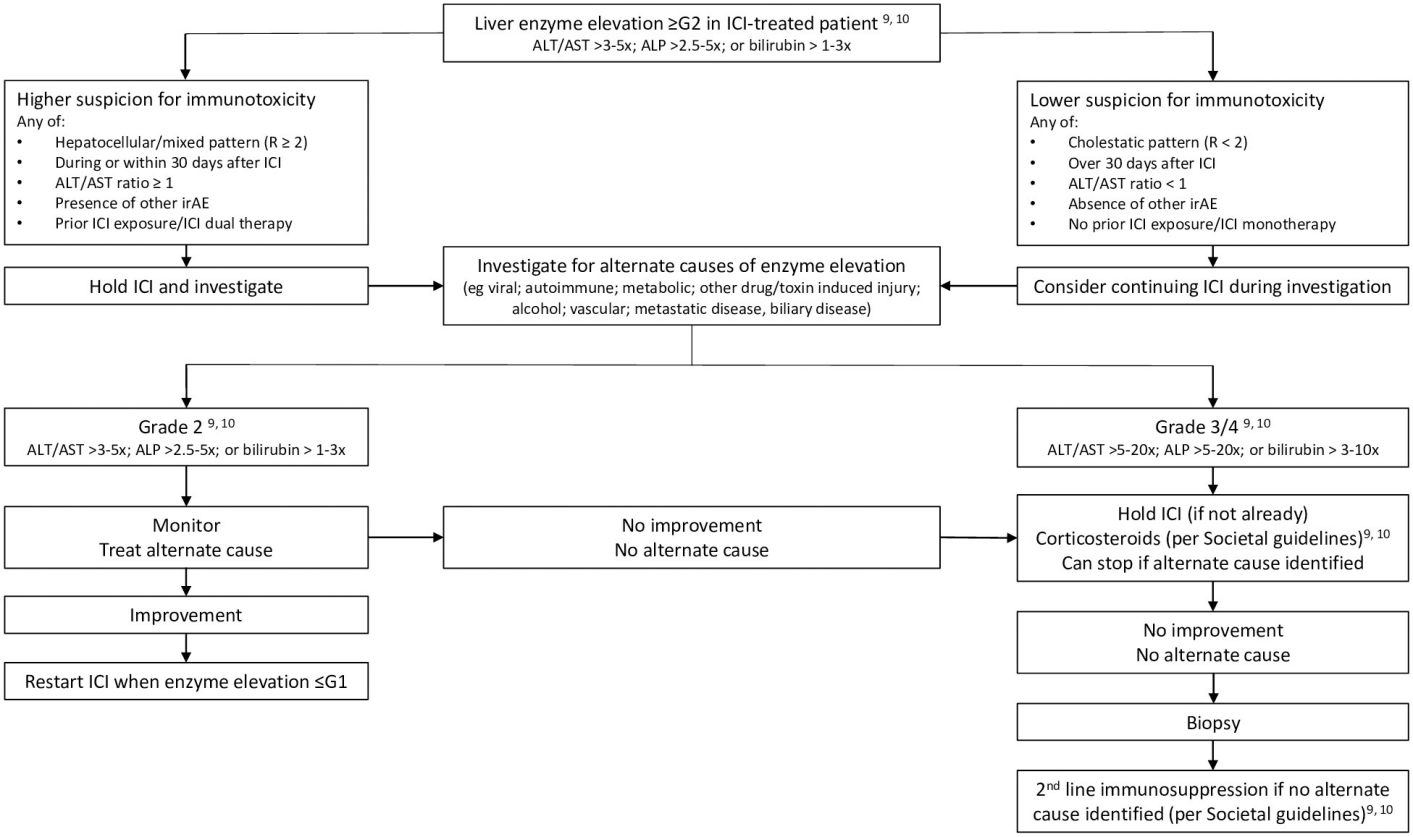
Proposed algorithm to guide diagnostic evaluation of immune checkpoint inhibitor (ICI)-treated patients with liver enzyme elevation. Stratification of ALT, AST, ALP and bilirubin elevation is as per CTCAE (v. 5.0; S1 Table) [6]. Definitions of Grade 2, 3 and 4 enzyme elevation as they relate to management of ICI-associated hepatotoxicity are as per European Society for Medical Oncology and American Society of Clinical Oncology Practice Guidelines [9, 10]. ALT, alanine transaminase, AST, aspartate aminotransferase; ALP, alkaline phosphatase.

Based on our findings, and other published literature, we propose an algorithm for diagnostic evaluation of liver enzyme elevations in ICI-treated patients (Fig 4). As most liver enzyme elevations will have another cause, in patients where the liver injury is mild and index of suspicion for CPI-mediated immunotoxicity is low, ICI could be continued during investigation, although liver enzymes should be monitored closely and this decision reviewed if liver injury progresses and/or investigations do not reveal an alternate cause for enzyme elevation. Given that ICI immunotoxicity can rarely cause fulminant liver failure [20, 21], initiation of treatment should not be delayed pending investigations in patients with suggestive clinical features and/or more severe liver injury (G3/4). If liver enzymes do not respond promptly, and initial non-invasive investigations are negative, then liver biopsy should be considered to exclude an alternate cause and confirm histopathological features consistent with immune-mediated hepatitis, before considering escalation to second line immunosuppression [9, 10].

There are a number of limitations to this study. The retrospective design is associated with inherent bias, although use of data which was prospectively collected in well-characterised clinical trial populations mitigates against this somewhat. These highly selected, closely monitored patients provided a very well-characterised cohort but are not necessarily representative of the wider population of patients treated with ICI. In particular, patients with pre-existing significant liver disease, or viral hepatitis, were excluded from these studies. The number of instances diagnosed as liver immunotoxicity was small, limiting evaluation and analysis. Classification of events as ICI toxicity was based on clinician judgement and cannot be fully verified retrospectively. Data and investigations outside our institution could not be accessed due to privacy regulations. Finally, management of liver enzyme elevations and suspected immunotoxicity was at the treating clinician’s discretion, following individual study protocol guidelines, leading to some heterogeneity in treatment.

In conclusion, this study demonstrates that liver enzyme elevations are common in patients with advanced metastatic cancer treated with ICI therapy. A full and prompt diagnostic evaluation is important to identify causes which may be unrelated to immunotherapy. Immunotoxicity is more likely to be associated with a mixed/hepatitic pattern of enzyme elevation, preserved ALT/AST ratio, other organ immunotoxicity and prior ICI exposure. We propose an algorithm to assist in the diagnostic evaluation of enzyme elevations in ICI-treated patients, however further prospective studies are required to refine diagnosis and management of ICI-mediated hepatotoxicity.

## Supporting information

S1 Table Common Terminology for Common Adverse Events (CTCAE) classification for bilirubin and liver enzyme elevations. **A**, version 5.0, dated November 2017. **B**, version 4.03, dated June 2010. ALT, alanine aminotransferase; AST, aspartate aminotransferase; ALP, alkaline phosphatase; ULN, upper limit of normal.
(PDF)

S2 Table Classes of investigational immunotherapy treatments received by study participants. CTLA-4 refers to drugs targeting anti-cytotoxic T-lymphocyte-associated protein 4; PD-1 refers to drugs targeting anti-programmed death protein 1; PD-L1 refers to drugs targeting anti-programmed death-ligand 1.
(PDF)

S3 Table List of primary malignancies of all study participants. HPB, hepato-pancreato-biliary; RCC, renal cell carcinoma; CNS, central nervous system; SCC, squamous cell carcinoma.
(PDF)

S4 Table Principal characteristics and treatment of patients diagnosed with liver immunotoxicity. ID; patient identifier for this study; ICI, immune checkpoint inhibitor; ALT, alanine aminotransferase; ALP, alkaline phosphatase; PD-1, programmed death protein 1; CTLA-4, cytotoxic T-lymphocyte-associated protein 4; PD-L1, programmed death-ligand 1; RCC, renal cell carcinoma; MMF, mycophenolate mofetil; NT, not tested.
(PDF)

S5 Table Range of other organ systems affected by other immune related adverse events (irAE) in patients with liver immunotoxicity, and timing of other irAE in relation to occurrence of liver immunotoxicity. (PDF)

S1 Fig Comparison of RUCAM scores between patients with liver enzyme elevations diagnosed with immune checkpoint inhibitor (ICI) associated hepatotoxicity (“immunotoxicity”; N = 17) and other causes for enzyme elevation (“other”; N = 103). Diagnoses were made contemporaneously and documented as the final diagnosis for enzyme elevation in the patient clinical record, by the treating physician. RUCAM scores were calculated retrospectively from information available in the clinical record, by an expert hepatologist (MC). RUCAM, Roussel Uclaf Causality Assessment Method.
(TIF)
