# Supplementary material for: Evaluation of liver enzyme elevations and hepatotoxicity in patients treated with checkpoint inhibitor immunotherapy
Source: PLoS One. 2021 Jun 11;16(6):e0253070. doi: 10.1371/journal.pone.0253070 (PMC8195413; doi:10.1371/journal.pone.0253070)
Supplement: S1 Table — A, version 5.0, dated November 2017. B, version 4.03, dated June 2010. ALT, alanine aminotransferase; AST, aspartate aminotransferase; ALP, alkaline phosphatase; ULN, upper limit of normal. (PDF) [file pone.0253070.s001.pdf]

A

**CTCAE v. 5.0**	**Total bilirubin**	**ALT/AST**	**ALP**
Grade 2	1.5 - 3 x ULN; 1.5 – 3 x baseline if baseline was abnormal	3 - 5 x ULN; 3 - 5 x baseline if baseline was abnormal	2.5 – 5 x ULN; 2.5 – 5 x baseline if baseline was abnormal
Grade 3	>3 - 10 x ULN; >3 - 10 x baseline if baseline was abnormal	>5 - 20 x ULN; 5 - 20 x baseline if baseline was abnormal	>5 - 20 x ULN; 5 - 20 x baseline if baseline was abnormal
Grade 4	>10 x ULN; >10 x baseline if baseline was abnormal	>20 x ULN; >20 x baseline if baseline was abnormal	>20 x ULN; >20 x baseline if baseline was abnormal

B

**CTCAE v. 4.03**	**Total bilirubin**	**ALT/AST**	**ALP**
Grade 2	1.5 - 3 x ULN	>3 - 5 x ULN	>2.5 – 5 x ULN
Grade 3	>3 - 10 x ULN	>5 - 20 x ULN	>5 - 20 x ULN
Grade 4	>10 x ULN	>20 x ULN	>20 x ULN
