# Supplementary material for: Evaluation of liver enzyme elevations and hepatotoxicity in patients treated with checkpoint inhibitor immunotherapy
Source: PLoS One. 2021 Jun 11;16(6):e0253070. doi: 10.1371/journal.pone.0253070 (PMC8195413; doi:10.1371/journal.pone.0253070)
Supplement: S2 Table — CTLA-4 refers to drugs targeting anti-cytotoxic T-lymphocyte-associated protein 4; PD-1 refers to drugs targeting anti-programmed death protein 1; PD-L1 refers to drugs targeting anti-programmed death-ligand 1. (PDF) [file pone.0253070.s002.pdf]

Treatment	Number of participants
CTLA-4 monotherapy	4
PD-1 monotherapy	206
PD-L1 monotherapy	78
CTLA-4 + PD-1	10
CTLA-4 +/- PD-1	3
CTLA-4 + PD-L1	14
PD-1 + PD-L1	6
CTLA-4 + other investigational immunotherapy	2
PD-1 + other investigational immunotherapy	107
PD-L1 + other investigational immunotherapy	38
CTLA-4 + PD-1 + other investigational immunotherapy	2
