# Supplementary material for: Evaluation of liver enzyme elevations and hepatotoxicity in patients treated with checkpoint inhibitor immunotherapy
Source: PLoS One. 2021 Jun 11;16(6):e0253070. doi: 10.1371/journal.pone.0253070 (PMC8195413; doi:10.1371/journal.pone.0253070)
Supplement: S3 Table — HPB, hepato-pancreato-biliary; RCC, renal cell carcinoma; CNS, central nervous system; SCC, squamous cell carcinoma. (PDF) [file pone.0253070.s003.pdf]

**Primary malignancy**	**Number of patients (% of total, N = 450)**
Melanoma	72 (16.0)
Head and neck	71 (15.8)
Genitourinary	70 (15.6)
Lung	60 (13.3)
Gastrointestinal	59 (13.1)
Breast	33 (7.3)
Musculoskeletal	28 (6.2)
HPB	25 (5.6)
RCC	16 (3.6)
CNS	6 (1.3)
Adenocarcinoma of unknown primary	3 (0.7)
Adrenal	1 (0.2)
Carcinoid	1 (0.2)
Merkel cell carcinoma	1 (0.2)
Neuroendocrine	1 (0.2)
Phaeochromocytoma	1 (0.2)
Dermal SCC	1 (0.2)
Eccrine carcinoma	1 (0.2)
