# Supplementary material for: Evaluation of liver enzyme elevations and hepatotoxicity in patients treated with checkpoint inhibitor immunotherapy
Source: PLoS One. 2021 Jun 11;16(6):e0253070. doi: 10.1371/journal.pone.0253070 (PMC8195413; doi:10.1371/journal.pone.0253070)
Supplement: S4 Table — ID; patient identifier for this study; ICI, immune checkpoint inhibitor; ALT, alanine aminotransferase; ALP, alkaline phosphatase; PD-1, programmed death protein 1; CTLA-4, cytotoxic T-lymphocyte-associated protein 4; PD-L1, programmed death-ligand 1; RCC, renal cell carcinoma; MMF, mycophenolate mofetil; NT, not tested. (PDF) [file pone.0253070.s004.pdf]

ID	Age (years) Sex	Primary	ICI	Prior ICI	Time to onset (days)	Peak ALT (IU/mL)	Peak ALP (IU/mL)	ANA; SMA; IgG	RUCAM	Steroid	Time to resolution (days)
LT1	63.5<br>F	Head and neck	PD-1	None	42	180	1189	Neg<br>NT<br>NT	6	Dexamethasone 4mg	20
LT2	47.7<br>M	Head and neck	PD-1	PD-1	295	1320	206	Pos<br>Neg<br>11.6	7	Steroid 1.5mg/kg iv	21
LT3	52.1<br>F	Melanoma	CTLA-4	None	57	220	251	NT<br>NT<br>NT	8	Prednisone 1mg/kg	62
LT4	36<br>F	Melanoma	PD-1	CTLA-4	62	78	71	NT<br>NT<br>9.5	5	None	7
LT5	53<br>F	Melanoma	CLTA-4	PD-1	23	287	229	NT<br>NT<br>10.1	9	Prednisone 1mg/kg	47
LT6	35.7<br>M	Melanoma	PD-1	CTLA-4	747	290	75	NT<br>NT<br>8.7	3	Prednisone taper	37
LT7	56<br>F	Melanoma	PD-1	PD-L1	50	414	358	Neg<br>Neg<br>22.1	9	Prednisone 1mg/kg	28
LT8	37<br>M	RCC	CTLA-4/PD-1	None	42	222	148	NT<br>NT<br>NT	4	Prednisone taper	77
LT9	77.5<br>M	Melanoma	PD-1	None	399	279	371	NT<br>NT<br>6.3	5	Steroid 2mg/kg iv	49
LT10	63.8<br>M	Pancreas	PD-1	None	25	132	291	Neg<br>NT<br>NT	3	Prednisone 1mg/kg	36
LT11	24<br>M	Colorectal	PD-1	None	274	370	356	NT<br>NT<br>NT	4	Steroid 1mg/kg iv	15
LT12	64.4<br>M	Head and neck	PD-1 or PD-1/ CTLA-4	None	99	364	193	Neg<br>Neg<br>5.1	9	Steroid 2mg/kg iv + MMF	48
LT13	18.9<br>F	Sarcoma	PD-1	None	78	311	69	Neg<br>Neg<br>5.9	8	Steroid 2mg/kg iv	98
LT14	75.4<br>M	Head and neck	PD-L1	None	39	110	91	NT<br>NT<br>NT	8	None	28
LT15	47.3<br>M	Melanoma	CTLA-4	PD-1	34	166	80	NT<br>NT<br>NT	2	Prednisone 1mg/kg	43
LT16	59.5<br>F	Colorectal	PD-1	None	95	36	158	NT<br>NT<br>NT	2	Prednisone 1mg/kg	102
LT17	53.4<br>F	Melanoma	PD-1	PD-1	44	160	54	NT<br>NT<br>NT	5	Prednisone 1mg/kg	12
