# Supplementary material for: Evaluation of liver enzyme elevations and hepatotoxicity in patients treated with checkpoint inhibitor immunotherapy
Source: PLoS One. 2021 Jun 11;16(6):e0253070. doi: 10.1371/journal.pone.0253070 (PMC8195413; doi:10.1371/journal.pone.0253070)
Supplement: S5 Table — (PDF) [file pone.0253070.s005.pdf]

Other irAE (N = 13)	Before liver immunotoxicity	Concurrent with liver immunotoxicity	After liver immunotoxicity
Endocrine	3		
Skin	2	1	
GI	2	2	
Respiratory	1	1	1
Renal			1
CNS			1
Musculoskeletal		2	
Haematology		1	
**Total**	**8**	**7**	**3**
