# Supplementary figures and images for: Evaluation of liver enzyme elevations and hepatotoxicity in patients treated with checkpoint inhibitor immunotherapy
Source: PLoS One. 2021 Jun 11;16(6):e0253070. doi: 10.1371/journal.pone.0253070 (PMC8195413; doi:10.1371/journal.pone.0253070)

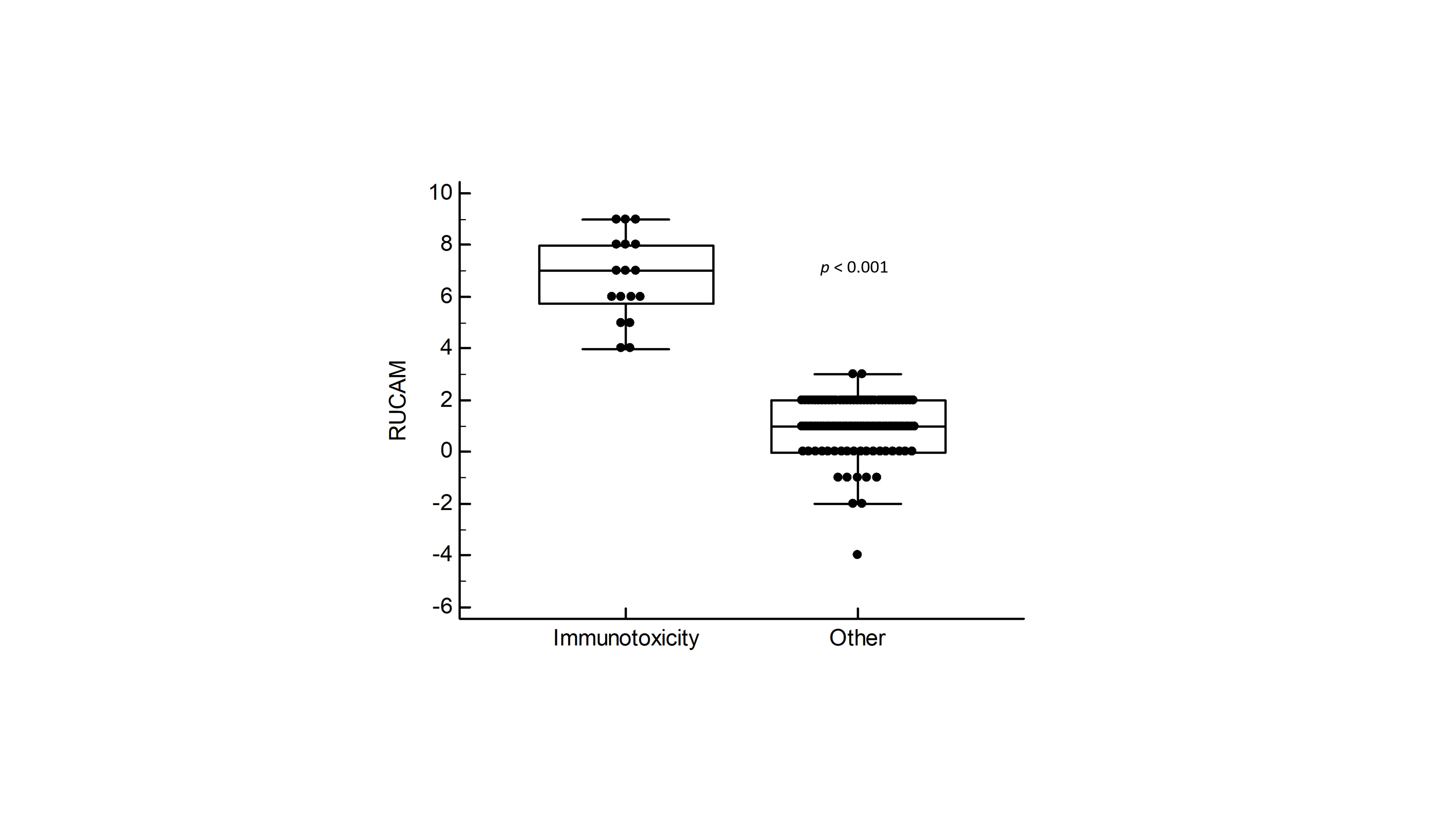

Supplement: S1 Fig — Diagnoses were made contemporaneously and documented as the final diagnosis for enzyme elevation in the patient clinical record, by the treating physician. RUCAM scores were calculated retrospectively from information available in the clinical record, by an expert hepatologist (MC). RUCAM, Roussel Uclaf Causality Assessment Method. (TIF) [file pone.0253070.s006.tif]
